# Epigenetic Regulation of Hepatic Stellate Cell Activation and Macrophage in Chronic Liver Inflammation

**DOI:** 10.3389/fphys.2021.683526

**Published:** 2021-07-01

**Authors:** Chun-xia Shi, Yao Wang, Fang-zhou Jiao, Qian Chen, Pan Cao, Mao-hua Pei, Lu-yi Zhang, Jin Guo, Wei Deng, Lu-wen Wang, Zuo-jiong Gong

**Affiliations:** Department of Infectious Diseases, Renmin Hospital of Wuhan University, Wuhan, China

**Keywords:** epigenetic regulation, macrophage, hepatic stellate cell, liver, inflammation

## Abstract

Chronic liver inflammation is a complex pathological process under different stress conditions, and the roles of stellate cells and macrophages in chronic liver inflammation have been widely reported. Moderate liver inflammation can protect the liver from damage and facilitate the recovery of liver injury. However, an inflammatory response that is too intense can result in massive death of hepatocytes, which leads to irreversible damage to the liver parenchyma. Epigenetic regulation plays a key part in liver inflammation. This study reviews the regulation of epigenetics on stellate cells and macrophages to explore the new mechanisms of epigenetics on liver inflammation and provide new ideas for the treatment of liver disease.

## Introduction

Most chronic liver diseases are accompanied by hepatic inflammation, which is a complex physiological and pathological process in response to various pressure conditions (Kubes and Mehal, 2012). Hepatic stellate cells (HSCs) and macrophages are important cells in chronic liver inflammation. Similar to most other organs, a certain degree of liver inflammation is needed to protect the liver from damage and facilitate the recovery of liver injury. However, an overly intense or chronic inflammatory response almost always leads to a massive loss of hepatocytes, which leads to severe liver damage (Schattenberg et al., 2006). Patients with severe liver function impairment and other organ failures usually show a strong systemic inflammatory response, which is correlated with the severity of the disease. When chronic inflammation stimulates fibroblasts to replace necrotic hepatocytes, liver fibrosis occurs and liver function also declines (Iwaisako et al., 2012). Since the liver is the main site for the production of complement, the decreased liver function reduces the synthesis of complement in serum, affects the immune function of the body, and further aggravates liver inflammation. Dysregulation of the inflammatory response is closely associated with liver injuries including (1) bacterial and viral infections; (2) poisoning by exogenous organisms or heavy metals; and (3) systemic metabolic diseases, such as obesity, diabetes, and metabolic syndrome (Navab et al., 2008). In this study, we review the epigenetic regulation on liver inflammation at the cellular level, focusing on the cellular and molecular mechanisms that trigger this phenomenon.

## Introduction of Epigenetic Modifications

Epigenetic regulation has a variety of ways. In this study, we mainly describe three ways of epigenetic regulation, namely, DNA methylation, expression of non-coding RNAs (ncRNAs), and histone modification.

## Non-Coding RNAs

Not all transcriptional genomes are translated into proteins, but they perform regulatory functions in the form of ncRNAs (Rinn and Chang, 2012). MicroRNAs (miRNAs) are the most thoroughly studied and have the most comprehensive functions so far. MiRNAs generally regulate target proteins by regulating the expression of messenger RNA (mRNA), and many miRNAs have been identified as targets for the treatment of diseases (Hassanein and Frederick, 2004; Janssen et al., 2013). Thus, there are more and more reports on long non-coding RNAs (lncRNAs), their lengths are >200 nucleotides, and they can be further divided into long intergenic RNAs (lincRNAs), intronic lncRNAs, and antisense lncRNAs. LncRNAs are considered to modulate various processes involved in liver diseases.

It has been reported that the transmission of ncRNAs can be mediated by exosomes. Exosomes are small extracellular vesicles (sEVs), 30–150 nm in diameter, that have been discovered in recent years. Almost all types of cells can release exosomes under physiological and pathological conditions. Exosomes play a crucial role in epigenetic regulation by transporting key molecules, such as miRNAs, lncRNAs, and proteins.

## Histone Modification and Chromatin Remodeling

Histone modification is regulated by histone-modifying enzymes (HMEs) (Hoeksema and de Winther, 2016). HME targets specific histones by interacting with transcription factors, and the modified histones can affect the density of chromatin structure and thus regulate gene expression. Histone modification is highly dynamic. Amino acids (AAs) in histone can be modified by methylation, phosphorylation, acetylation, and ubiquitination. Histone acetylation loosens the chromatin structure and promotes transcription, while histone deacetylases (HDACs) deacetylate AA residues in histone, which makes histone bind more closely to negatively charged DNA, leading to chromatin concentration and inhibiting gene transcription. The trimethylation of histone 3 lysine 9 (H3K9me3) can also concentrate chromatin and suppress transcription. However, not all modifications of lysine methylation inhibit transcription, for example, H3K4me3 usually promotes transcription. H3K4me3 is a sign of genetic start, and H3K4me1 is usually associated with the remote control components (enhancement). Besides, H3K27ac near transcription start site or on the enhancer and H3K36me3 throughout the genome both usually promote gene transcription.

## DNA Methylation

DNA methylation is regulated by DNA methyltransferases (DNMTs) and ten-eleven translocation (TET) enzymes. DNMT converts cytosine to 5-methylcytosine that is bound to guanine (Iacobazzi et al., 2013), while the TET enzyme catalyzes progressive oxidation of methyl and ultimately reduces unmodified cytosine residues (Tahiliani et al., 2009). The most well-known modification of DNA is the methylation of cytosine at its fifth carbon ring, which is common within the cytosine–phosphate–guanine (CpG) dinucleotide, and the methylation of CpG leads to intense transcriptional suppression. Most methylated CpGs develop stably in adults. In cancer, however, CpG methylation is usually accompanied by an oppressive epigenetic marker, the methylation of H3K27, which concentrates chromatin and suppresses transcription (Suganuma and Workman, 2011).

## Epigenetic Regulation of Hepatic Stellate Cell and Macrophage in Chronic Liver Inflammation

Hepatic stellate cells and macrophages are two important cell groups in the process of chronic liver inflammation, but their roles in liver injury are different. After the injury of hepatocytes, macrophages are activated to produce cytokines and signaling molecules, including tumor necrosis factor alpha (TNF-α), interleukin 1 beta (IL-1β), interleukin 6 (IL-6), and other signaling molecules (Seki et al., 2001), which activate downstream pathways and clear pathogens. While fibrosis occurs in the liver, HSC is activated. The activation of HSC comes from the inflammatory activity of liver immune cells, mainly macrophages (Koyama and Brenner, 2017). For example, damaged hepatocytes activate Kupffer cells, resulting in the release of IL-1β, thereby inducing the activation of HSC (Miura et al., 2010). When there is an occurrence of inflammation in the liver (including fibrosis), macrophages are activated, but HSC is mainly involved in the occurrence of liver fibrosis. The epigenetic regulation of chronic liver inflammation by HSC and macrophages was discussed separately.

## Hepatic Stellate Cell

### Non-Coding RNAs

In the case of liver injury, especially chronic inflammation, HSCs are activated and transdifferentiated into hepatic myofibroblasts to repair wounds, and miRNA can regulate the posttranscriptional expression of HSC. Analysis of miRNA arrays in human HSC isolated from normal liver revealed a small number of miRNAs expressed in these cells, 47 of which were downregulated after activation, while 212 were upregulated (Coll et al., 2015). In epigenetic signaling, miRNA can regulate the activation of HSC (Yu et al., 2020). The fibrosis-promoting miRNAs include miR-873-5p (Fernandez-Ramos et al., 2018), miR-21 (Zhang et al., 2013), miR-942 (Tao et al., 2018), miR-125b (You et al., 2018), miR-221 and miR-222 (Ogawa et al., 2012), and miR-27 (Ji et al., 2009), while the anti-fibrosis miRNAs include miR-200a (Yang J. J. et al., 2014, 2017), miR-101-3p (Meroni et al., 2019), miR-214 (Chen et al., 2014), miR-378a (Hyun et al., 2016; Yu et al., 2016), miR-148a (Liu et al., 2015; Jung et al., 2016), miR-29 (Huang et al., 2015; Yang Y. L. et al., 2017, 2019), miR-15b and miR-16 (Guo et al., 2009), miR-122 (Li et al., 2013), miR-133a (Roderburg et al., 2013), miR-195 (Sekiya et al., 2011), miR-150 and miR-194 (Venugopal et al., 2010; Chen W. et al., 2020), and miR-30, miR-335, let-7, and miR-338 (Winkler et al., 2020). The mechanism of miRNAs regulating HSC activation is complex. Different miRNAs regulate activation of HSC and liver fibrosis through targeting different molecules or pathways. In addition to regulating some signaling pathways, miRNAs also act on some histone-modifying enzymes, such as HDAC4, Sirtuin 1(SIRT1) (a class III HDAC), histone methyltransferase DNMT, and glycine N-methyltransferase (GNMT) (Huang et al., 2015; Yang J. J. et al., 2017; Yang Y. L. et al., 2017, 2019; Fernandez-Ramos et al., 2018). The specific mechanisms by which miRNAs regulate liver fibrosis are summarized in Supplementary Table 1.

Long non-coding RNAs (LncRNAs) can also regulate the activation of HSC. LncRNAs regulate gene expression by competing with miRNAs or acting as miRNA sponges. For example, lncRNA-p21 is increased significantly in liver fibrosis and regulates miR-30 by acting as a competing endogenous RNA (ceRNA), and suppression of miR-30 can weaken the effect of lncRNA-p21 on fibrosis (Tu et al., 2017). Similar mechanisms include the negative regulation of lncRNA HOX transcript antisense RNA (HOTAIR) on miR-29 (Yang Y. L. et al., 2017; Yu et al., 2017) and the regulation of lncRNA plasmacytoma variant translocation 1 (PVT1) on miR-152 (Zheng et al., 2016). Both HOTAIR and PVT1 can activate HSC and promote liver fibrosis. LncRNA H19 can also stimulate liver fibrosis, the expression of which is increased in human and mouse liver at fibrosis (Chen et al., 2015; Zhang et al., 2016). However, other studies reported that lncRNA H19 expression was decreased in activated HSC and fibrotic liver tissues of rats (Yang J. J. et al., 2016, 2018). The decreased expression of lncRNA H19 may be due to the expression of histone methyltransferase DNMT1 and increased methylation in the H19 promoter region. Knocking down DNMT1 can increase the expression of H19 in activated HSC (Yang J. J. et al., 2018). Therefore, the regulation of lncRNA H19 on HSC may have a more complex mechanism. The expression of lncRNA antisense non-coding **RNA** in the INK4locus (ANRIL) is also decreased in liver fibrosis tissues and activated HSC, and the decreased expression of ANRIL is associated with histone methyltransferase DNMT3A (Yang J. J. et al., 2020). CircRNAs are involved in the activation of HSC as well, for example, mmu_circ_34116, mmu_circ_33594, and mmu_circ_35216 are significantly increased in mouse HSC cell lines and fibrotic liver tissues (Zhou Y. et al., 2018). These results reveal that ncRNAs play a crucial part in the activation of HSC, and an in-depth understanding of the mechanism of their action may provide new ways for the treatment of liver fibrosis.

Recent studies have shown that exosomes are involved in the activation of HSC and that exosomes play an important role in the communication between hepatocytes, macrophages, and HSCs. Exosomes mediate the communication between hepatocytes and HSC during hepatitis C virus (HCV) infection, and miR-19a in exosomes secreted by HCV-infected hepatocytes can activate HSCs and promote liver fibrosis (Devhare et al., 2017). Exosomes from palmitic acid (PA)-treated hepatocytes can also mediate communication between hepatocytes and HSC, promoting the activation of HSC (Lee et al., 2017). In addition, exosomal miR-103-3p from lipopolysaccharide (LPS)-activated THP-1 cells (a human leukemia monocytic cell line) can promote the proliferation of HSC and plays an important role in the communication between THP-1 macrophages and HSC during the progression of liver fibrosis (Chen L. et al., 2020). Exosomal lncRNA H19l derived from bile duct cells can also promote differentiation and activation of HSC and promote cholestatic liver fibrosis (Liu et al., 2019). In a nutshell, the role of exosomes in the activation of HSC has been confirmed by an increasing number of reports. The mechanisms of lncRNAs on HSC are summarized in Supplementary Table 2.

### DNA Methylation

Significant changes in global DNA methylation are observed during the activation of HSC, and methyl donors are also related to HSC activation, revealing that DNA methylation plays a key role in the activation of HSC (Gotze et al., 2015; Page et al., 2016; Cheng et al., 2020; Zhu et al., 2020). Usually, gene expression is inhibited by methylation of CpG dinucleotide in the promoter region, which is regulated by DNMTs (Hardy and Mann, 2016). Reports have shown that methyl-CpG-binding protein 2 (MeCP2) is highly expressed during the transdifferentiation of HSC and plays an important role in the activation of HSC (Bian et al., 2013). MeCP2 activates HSC by negatively regulating the two key molecules inhibitor of nuclear factor kappa B alpha (IκBα) and peroxisome proliferator-activated receptor gamma (PPARγ) that maintain the stasis phenotype of HSC. The mechanism by which MeCP2 negatively regulates IκBα and PPARγ is mainly through interaction with the promoter; for example, MeCP2 is recruited to the promoter region of PPARγ, promoting H3K9 methylation and recruiting the transcriptional repressor **heterochromatin protein 1** alpha (HP1α). DNA methylation inhibitor 5-aza-2′-deoxycytidine (5-azadC) prevents the loss of IκBα and PPARγ, which can maintain the stasis phenotype of HSC (Mann et al., 2007, 2010). MeCP2 also negatively regulates lncRNA H19 to promote the proliferation of HSC (Yang et al., 2013; Yang J. J. et al., 2016). The team further discovered that HSC transdifferentiation is regulated by MeCP2, histone methyltransferase **enhancer of zeste homolog** 2 (EZH2), and miR-132. MiR-132 negatively regulates MeCP2 (miR-132 expression is absent in HSC during liver injury), and MeCP2 promotes EZH2 expression and H3K27 methylation (Mann et al., 2010). These results show direct evidence for epigenetic regulation on the activation of HSC. The mechanisms of MeCP2 on HSC are summarized in Supplementary Table 3.

### Histone Modification

Histone methylation is catalyzed by histone methyltransferases, such as EZH2, myeloid/lymphoid or mixed-lineage leukemia 1 (MLL1), absent, small, or homeotic discs 2 (ASH2), and WD repeat-containing protein 5 (WDR5), and histone demethylases (KDMs), including lysine-specific demethylase (LSD) family and jumonji C domain-containing (JMJD) family. During HSC activation, histone methyltransferases are recruited, such as ASH2, WDR5, and EZH2 (Mann et al., 2010; Kong et al., 2019a). Histone methyltransferase and demethylase have opposite effects on the activation of HSC. For example, methyltransferase EZH2 promotes HSC activation and fibrosis by inhibiting the transcription of PPARγ (Mann et al., 2010), while histone H3K9 demethylase JMJD1A can promote PPARγ expression by regulating the demethylation of PPARγ gene and thus inhibit HSC activation and fibrosis (Jiang et al., 2015). Inhibition of EZH2 can decrease H3K27me3 on the genes coding anti-inflammatory cytokines, promote gene expression, and inhibit the activation of HSC, whereas inhibition of JMJD3 has the opposite effect. EZH2 inhibitor and JMJD3 activator have the potential to be the new direction of blocking liver fibrogenesis (Martin-Mateos et al., 2019; Jiang et al., 2021). Besides, during the activation of HSC, histone demethylase KDM4 is reduced, and KDM4 can suppress HSC activation by inducing the transcription of miR-29 (Kong et al., 2019b). KDM4D, a member of the KDM4 family, regulates HSC activation through toll-like receptor 4 or nuclear factor kappa-light-chain-enhancer of activated B (TLR4/NF-κB) signaling pathway (Dong et al., 2019).

**Figure 1:**
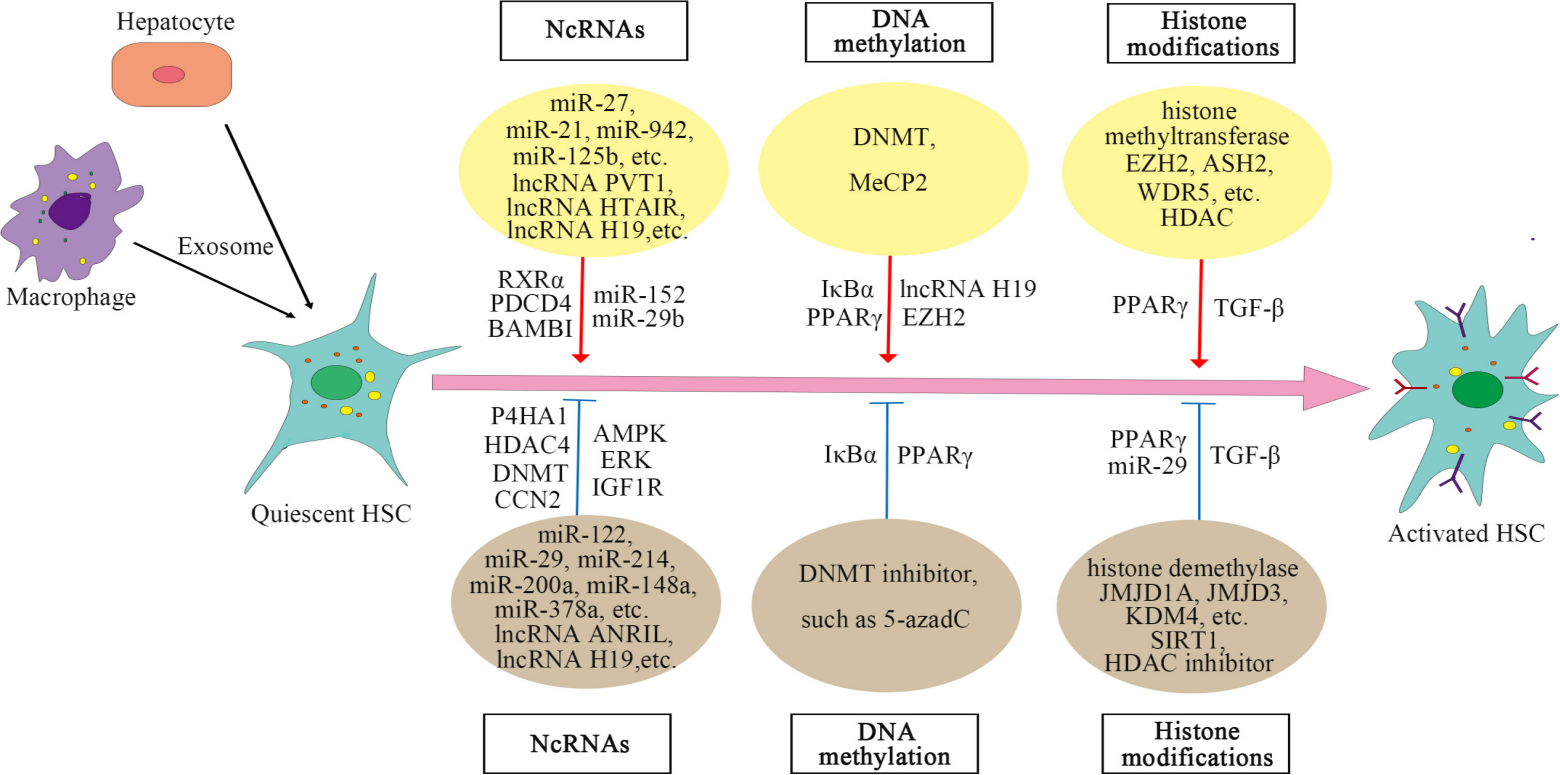
Epigenetic regulation of hepatic stellate cell (HSC) activation.

Acetylation is also critical for regulating gene expression. **Transforming growth factor-β** (TGF-β) is a key molecule that induces the activation of HSC. Histone acetyltransferase P300 can enhance the HSC response to TGF-β (Wang et al., 2016, 2019; Dou et al., 2018), whereas histone deacetylase SIRT1, contrary to P300, has a weakened effect on the TGF-β pathway and inhibits HSC transdifferentiation (Li et al., 2017, 2018; Jiang et al., 2019). Moreover, in liver fibrosis, high expression of HDAC can activate HSC by regulating miRNA transcription, while HDAC inhibitors can suppress the activation of HSC (Han et al., 2017; Yang Z. et al., 2017; Lu et al., 2019). For example, HDAC inhibitor suberoylanilide hydroxamic acid (SAHA) can improve liver fibrosis in rats (Wang et al., 2018). The mechanisms of HMEs on HSC are summarized in Supplementary Table 3. And the epigenetic regulation of HSC activation is shown in Figure 1.

## Macrophage

Hepatic macrophages mainly include Kupffer cells (resident liver macrophages) and circulating monocytes (van der Heide et al., 2019). Monocytes and macrophages are the key drivers of inflammation, and macrophages transform into two extreme phenotypes, classically activated phenotype (M1) and alternately activated phenotype (M2), under different stimuli (Gordon, 2003). However, the phenotype of macrophages is not limited to these two extreme phenotypes but to a continuous spectrum related to the function of macrophages (Colin et al., 2014; Orecchioni et al., 2019). Epigenetic regulation has been shown to be involved in the reprogramming of monocytes and macrophages and can regulate the transcription and phenotype of macrophages (Saeed et al., 2014; Amit et al., 2016; Zhou et al., 2017). For example, DNA methylation regulates polarization and activation of hepatic macrophages (Yang Y. et al., 2018; Jain et al., 2019), and miR-221 and miR-222 regulate macrophage functional reprogramming (Seeley et al., 2018). HDAC2 and HDAC3 can also regulate inflammatory genes in macrophages (Raghuraman et al., 2016).

### Non-Coding RNAs

Non-coding RNAs play a crucial part in regulating transcription in all aspects. For example, ncRNAs can promote or inhibit transcription by recruiting HME or transforming mRNA splicing (Katayama et al., 2005). NcRNAs, especially miRNAs, play a crucial role in regulating the function of macrophages. Studies have found that the expression of miR-155 is increased in Kupffer cells of alcoholic liver disease model mice. Further studies have shown that miR-155 can directly regulate the function of macrophages (Bala et al., 2017). MiR-155-deficient mice showed reduced expression of genes related to steatosis and fatty acid (FA) metabolism, and reduced steatosis and fibrosis in steatohepatitis (Csak et al., 2015). In addition, the expression of miR-142-5p increased, and miR-130a-3p decreased in macrophages from patients with cirrhosis. These changes can regulate the transcription of pre-fibroblast genes in macrophages and maintain the fibro-promoting effect of macrophages. Inhibition of miR-142-5p and increase in miR-130a-3p expression can inhibit chemokine (C-C motif) ligands 4 (CCL4)-induced liver fibrosis in mice (Su et al., 2015). Therefore, miRNAs are important regulators of inflammatory signals in liver macrophages.

Exosomal miRNAs also play an important role in regulating the function of macrophages in chronic liver inflammation. Reports have shown that the hepatitis B virus (HBV) can encode a miRNA (HBV-miR-3) that inhibits HBV replication by targeting HBV transcripts. HBV-miR-3 in exosomes can promote M1 macrophage polarization, and exosomes containing HBV-miR-3 can increase the secretion of IL-6, indicating exosomal HBV-miR-3 may inhibit hepatocyte damage caused by HBV replication through activating immune response (Zhao et al., 2020). The study also finds that the level of miRNA-122 in liver monocytes and Kupffer cells of alcohol-fed mice is increased, and exosomal miRNA-122 derived from hepatocytes can reprogram monocytes. The exosomes (containing miRNA-122) from Huh7.5 cells can be absorbed by THP-1 monocytes. The miRNA-122 transferred through exosomes further promotes the expression of inflammatory factors. These results indicate that exosomes can mediate the communication between hepatocytes and monocytes/macrophages and then affect the function of macrophages (Momen-Heravi et al., 2015).

### DNA Methylation

The regulation of DNA methylation on macrophages is mainly manifested in the regulation of methylation of inflammation-related genes, such as proline–serine–threonine phosphatase-interacting protein 2 (PSTPIP2), suppressor of cytokine signaling 1 (SOCS1), zinc finger swim-type containing 3 (ZSWIM3) genes, and so on (Cheng et al., 2014; Yang Y. et al., 2018; Li et al., 2020). Studies have found that CCL4 induces more DNA methylation on the CpG islands of liver macrophages in mice. Among the 26 liver fibrosis-related genes verified, 130 CpG sites in the CpG islands of the PSTPIP2 gene are significantly hypermethylated, and the expression of PSTPIP2 is significantly reduced (Yang Y. et al., 2018). *In vitro* experiments have found that the hypermethylation of PSTPIP2 is mediated by methyltransferases DNMT3a and DNMT3b. Further studies have found that PSTPIP2 overexpression can inhibit M1 macrophage polarization and promote M2 macrophage polarization (Yang Y. et al., 2018). These results indicate that the DNA methylation of PSTPIP2 in macrophages can affect liver inflammation and fibrosis in mice by regulating the polarization of macrophages. In addition, SOCS1 plays a key role in inhibiting tissue damage and inflammation. Knockout of DNMT1 or DNA methylation inhibitors to treat LPS-induced RAW264.7 macrophages can reduce the hypermethylation of SOCS1 promoter and upregulate the expression of SOCS1, thereby inhibiting the release of inflammatory factors such as TNF-α and IL-6 in macrophages (Cheng et al., 2014), indicating that DNMT1-mediated SOCS1 hypermethylation leads to the loss of SOCS1 expression and enhances the release of cytokines in macrophages. Another important gene is ZSWIM3, which has been reported to activate the NF-κB pathway and affect inflammatory response. Preliminary screening of macrophage methylation shows that ZSWIM3 is hypermethylated in the 5′-untranslated region (5′-UTR) and is consistently reduced in macrophages isolated from the liver of ethanol-fed mice (Li et al., 2020). The abnormal expression of ZSWIM3 in alcoholic liver injury (ALI) is related to its hypermethylation. DNMTs-small interfering RNA (siRNA) and methylation inhibitors can rescue downregulated ZSWIM3. Chromatin immunoprecipitation (ChIP) analysis shows that DNMT3b is the main regulator of ZSWIM3 (Li et al., 2020). These studies confirm the important role of DNA methylation in the function of macrophages.

### Histone Modification

Histone methylation is a widespread epigenetic marker. Histone methyltransferases EZH2 and H3K27me3 catalyzed by EZH2 are significantly upregulated in Kupffer cells of mice with liver failure, which can trigger the release of pro-inflammatory cytokines, such as TNF, and activate NF-κB and protein kinase B (Akt) signaling pathways to participate in the pathogenesis of liver failure. EZH2 inhibitors can relieve the severity of liver failure in mice, which may be related to the reduction in H3K27me3 and the downregulation of liver pro-inflammatory cytokines (Zhou T. et al. 2018). The methyl donor S-adenosylmethionine (SAMe) can also inhibit the activity of methyltransferase in Kupffer cells and further inhibit the promoter H3K4me3, thereby blocking LPS-induced TNF-α secretion and expression of inducible nitric oxide synthase (iNOS) in Kupffer cells (Ara et al., 2008). Furthermore, inhibition of PPARγ mediated by histone methyltransferase suppressor of variegation 3-9 homolog 2 (SUV39H2) in macrophages promotes pro-inflammatory M1 polarization, thereby promoting liver inflammation. Lack of SUV39H2 protects mice from non-alcoholic steatohepatitis (Fan et al., 2017).

**Figure 2:**
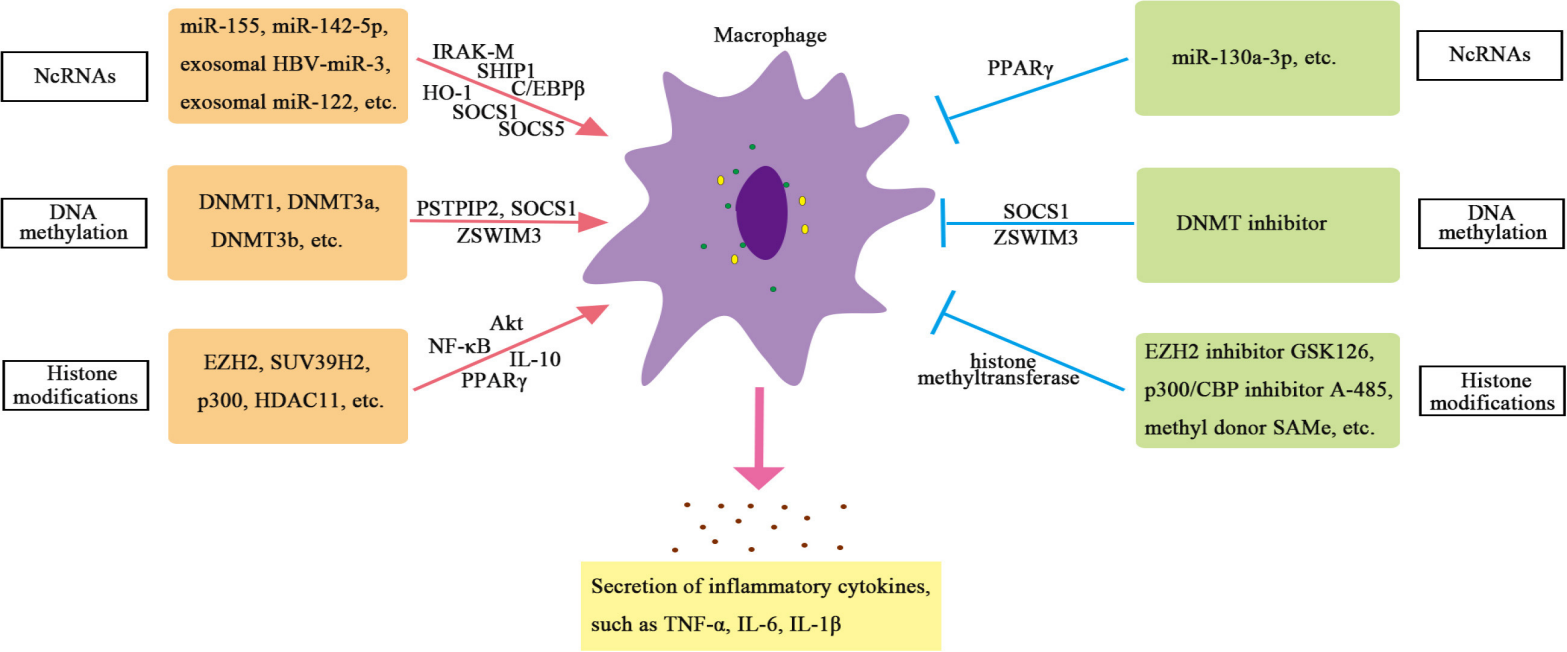
Epigenetic regulation of hepatic macrophage.

Compared with blood monocytes, Kupffer cells show significant levels of H3K27 acetylation (Sakai et al., 2019). When comparing Kupffer cells isolated from the liver of non-alcoholic steatohepatitis mice with Kupffer cells isolated from homeostasis mice, more than 6,000 enhancers are found to be significantly different in H3K27ac. Both histone acetylase and histone deacetylase are involved in regulating the role of macrophages in chronic liver inflammation. Studies have shown that histone acetylase P300 can regulate the polarization of macrophages, and knockdown of P300 or application of highly selective P300/CBP inhibitors can inhibit M1 polarization and significantly reduce the production of inflammatory factors during liver injury. The mechanism is that the expression of pro-inflammatory genes is inhibited by inhibiting H3K27/H3K18 acetylation in the promoter region of key inflammatory genes, leading to reduced activation of inflammatory pathway in the liver injury mice model, thus reducing M1 polarization and playing a protective role in the liver (Peng et al., 2019). Besides, histone acetyltransferase MOF is downregulated in human steatohepatitis. Liver injury induced by MOF deletion requires the interaction between hepatocytes and Kupffer cells. The loss of MOF in hepatocytes does not show obvious liver abnormalities. Only the loss of MOF in macrophages and hepatocytes at the same time can lead to enhanced expression of inflammatory genes and nitric oxide (NO) signal transduction, which further leads to hepatocyte apoptosis and lipotoxicity. These results indicate that the expression of histone acetyltransferase MOF in macrophages plays an important role in maintaining normal liver metabolism (Lei et al., 2020). In addition, histone deacetylase 11 (HDAC11) is induced in Kupffer cells of alcoholic liver disease model mice, which reduces the expression of IL-10. Knocking out HDAC11 results in an increase in IL-10 expression and a decrease in TNF secretion in macrophages, which suggests an important role of HDAC11 in promoting inflammation in macrophages (Bala et al., 2017). Therefore, the regulation of HMEs on macrophages is important and complex. The mechanisms of epigenetic regulation on hepatic macrophages are summarized in Supplementary Table 4 and displayed in Figure 2.

## Summary And Prospect

Hepatic stellate cell (HSC) is activated during chronic liver inflammation to repair liver tissue damage, and macrophages play a crucial part in inflammation. Transcription factors modify chromatin during stimulation and form cellular memory, enabling cells to respond more quickly when restimulated. Although this regulation allows inflammatory cells to respond effectively to external stimuli, it is also prone to dysregulation. Mild inflammation can result in changes in the status of inflammatory cells, which are driven by cytokine-induced epigenetics. In conclusion, the influences of transcription factors and epigenetics on the activation of inflammation-related cells play an indispensable part in the pathogenesis of chronic liver inflammatory diseases, which will be a potential target for liver inflammatory diseases in the future.

## Author Contributions

C-xS designed and wrote the paper. YW, F-zJ, QC, and PC performed the literature search. M-hP, L-yZ, JG, WD, and L-wW contributed to the research and discussion of content. Z-jG contributed to supervision and reviewed the manuscript. All authors contributed to the article and approved the submitted version.

## Conflict of Interest

The authors declare that the research was conducted in the absence of any commercial or financial relationships that could be construed as a potential conflict of interest.
