# Supplementary material for: Epigenetic Regulation of Hepatic Stellate Cell Activation and Macrophage in Chronic Liver Inflammation
Source: Front Physiol. 2021 Jul 1;12:683526. doi: 10.3389/fphys.2021.683526 (PMC8281248; doi:10.3389/fphys.2021.683526)
Supplement: Supplementary file 1 [file Table_1.DOC]

**Table 1 MiRNAs regulate fibrosis by different mechanisms**

| **MiRNAs** | **Effect on HSC** | **Targets** | **Cell types** |
| --- | --- | --- | --- |
| miR-873-5p | - | GNMT | mouse hepatocytes (Fernandez-Ramos et al., 2018) |
| miR-21 | activates | PDCD4/AP-1 | rat HSCs (Zhang et al., 2013) |
| miR-942 | activates | BAMBI | human HSCs and LX2 cells (Tao et al., 2018) |
| miR-125b | activates | Stard13/RhoA/α-SMA | mouse HSCs and JS1 cells (You et al., 2018) |
| miR-27 | activates | RXRα | rat HSCs (Ji et al., 2009) |
| miR-200a | inhibits | SIRT1/Notch1 | HSC-T6 cell lines (Yang et al., 2017) |
|  |  | KEAP1/Nrf2 | HSC-T6 cell lines (Yang et al., 2014) |
| miR-214 | inhibits | CCN2 | mouse HSCs (Chen et al., 2014) |
| miR-378a | inhibits | Gli3 | mouse HSCs and LX2 cells (Hyun et al., 2016) |
|  |  | TGF-β2 | LX2 cells and rat HSCs (Yu et al., 2016) |
| miR-148a | inhibits | IKKα/NUMB/NOTCH | Huh7 cell lines and HepaRG cells (Jung et al., 2016) |
|  |  | Gas1 | LX2 cells and HSC-T6 cells (Liu et al., 2015) |
| miR-29 | inhibits | HDAC4 | mouse HSCs (Huang et al., 2015) |
|  |  | DNMT3b | mouse HSCs (Yang et al., 2019) |
|  |  | DNMT1, DNMT3b, SET1A, PTEN | mouse HSCs (Yang et al., 2017) |
| miR-15b, miR-16 | inhibits | Bcl-2, caspase 3, 8, 9 | rat HSCs (Guo et al., 2009) |
| miR-122 | inhibits | P4HA1 | LX2 cells and rat HSCs (Li et al., 2013) |
| miR-133a | inhibits | Col1a1, Col5a3 | mouse HSCs (Roderburg et al., 2013) |
| miR-195 | inhibits | cyclin E1 | LX2 cells and mouse HSCs (Sekiya et al., 2011) |
| exosomal miR-19a | activates | SOCS/STAT3/TGF-β | LX2 cells (Devhare et al., 2017) |
| exosomal miR-103-3p | activates | KLF4 | LX2 cells (Chen et al., 2020) |
