# Supplementary material for: Epigenetic Regulation of Hepatic Stellate Cell Activation and Macrophage in Chronic Liver Inflammation
Source: Front Physiol. 2021 Jul 1;12:683526. doi: 10.3389/fphys.2021.683526 (PMC8281248; doi:10.3389/fphys.2021.683526)
Supplement: Supplementary file 2 [file Table_2.DOC]

**Table 2** LncRNAs regulate fibrosis by different mechanisms

| **LncRNAs** | **Effect on HSC** | **Targets** | **Cell types** |
| --- | --- | --- | --- |
| lncRNA-p21 | - | miR-30 | mouse hepatocytes (Tu et al., 2017) |
| lncRNA ANRIL | inhibits | AMPK | HSC-T6 cell lines (J. J. Yang et al., 2020) |
| lncRNA H19 | activates | - | Huh7 cells and Hepa1 cells (Y. Zhang et al., 2016) |
| lncRNA H19 | inhibits | ERK | HSC-T6 cell lines (J. J. Yang et al., 2018) |
|  |  | IGF1R | HSC-T6 cell lines (J. J. Yang et al., 2016) |
| lncRNA HOTAIR | activates | miR-29b/PTEN | mouse HSCs and hepatocytes (Yu et al., 2017) |
| lncRNA PVT1 | activates | miR-152/PTCH1 | mouse HSCs (Zheng et al., 2016) |
