# Supplementary material for: Epigenetic Regulation of Hepatic Stellate Cell Activation and Macrophage in Chronic Liver Inflammation
Source: Front Physiol. 2021 Jul 1;12:683526. doi: 10.3389/fphys.2021.683526 (PMC8281248; doi:10.3389/fphys.2021.683526)
Supplement: Supplementary file 3 [file Table_3.docx]

**Table 3** The mechanisms of epigenetics on HSCs

| **Epigenetic regulators** | **Effect on HSC** | **Targets** | **Cell types** |
| --- | --- | --- | --- |
| MeCP2 | activates | IκBα, PPARγ, EZH2, lncRNA H19/IGF1R | rat and mouse HSCs, LX2 cells, HSC-T6 cell lines (Mann et al., 2010; Mann et al., 2007; Yang et al., 2016; Yang et al., 2013) |
| EZH2 | activates | PPARγ | rat and mouse HSCs (Mann et al., 2010) |
| JMJD1A | inhibits | PPARγ | rat HSCs and LX2 cells (Jiang et al., 2015) |
| KDM4 | inhibits | miR-29 | mouse HSCs and LX2 cells (Kong et al., 2019) |
| P300 | activates | CXCL12, SMAD2/3, TAZ | mouse and human HSCs, LX2 cells (Dou et al., 2018; Wang et al., 2019) |
| SIRT1 | inhibits | Smads, EZH2, PPARγ | LX2 cells, HSC-T6 cell lines, mouse HSCs (Jiang et al., 2019; Li et al., 2018) |
