# Supplementary material for: Epigenetic Regulation of Hepatic Stellate Cell Activation and Macrophage in Chronic Liver Inflammation
Source: Front Physiol. 2021 Jul 1;12:683526. doi: 10.3389/fphys.2021.683526 (PMC8281248; doi:10.3389/fphys.2021.683526)
Supplement: Supplementary file 4 [file Table_4.docx]

**Table 4** The mechanisms and effects of epigenetics on hepatic macrophages

| **Epigenetic regulators** | **Targets** | **Effects on macrophage** | **Cell types** |
| --- | --- | --- | --- |
| miR-155 | IRAK-M, SHIP1, C/EBPβ | TNF-α secretion | Kupffer cells (Bala et al., 2017) |
| miR-142-5p | SOCS1 | fibrosis promotion | human macrophages (Su et al., 2015) |
| miR-130a-3p | PPARγ | fibrosis inhibition | human macrophages (Su et al., 2015) |
| exosomal HBV-miR-3 | SOCS5 | IL-6 secretion | HepG2 cells (Zhao et al., 2020) |
| exosomal miR-122 | HO-1 | TNF-α, IL-1β secretion | THP1 cells (Momen-Heravi et al., 2015) |
| DNMT3a, DNMT3b | PSTPIP2/STAT1, 6 | M1 polarization | mouse hepatic macrophages and RAW264.7 cells (Yang et al., 2017) |
| DNMT1 | SOCS1 | TNF-α, IL-6 secretion | RAW264.7 cells (Cheng et al., 2014) |
| DNMT3b | ZSWIM3/TRAF2/NF-κB | TNF-α, IL-6, IL-1β secretion | mouse hepatic macrophages and RAW264.7 cells (Li et al., 2020) |
| SUV39H2 | PPARγ | M1 polarization | Kupffer cells BMDMs (Fan et al., 2017) |
| SAMe | histone methyltransferase | TNF-α and iNOS inhibition | Kupffer cells (Ara et al., 2008) |
| p300 | H3K27/H3K18 ac | M1 polarization | RAW264.7 cells and primary BMDM cells (Peng et al., 2019) |
| HDAC11 | IL-10 | TNF secretion | Kupffer cells (Bala et al., 2017) |
